# Targeting gut microbiota and metabolites in cancer radiotherapy

**DOI:** 10.1002/ctm2.70481

**Published:** 2025-09-30

**Authors:** Shuling Ma, Xinpei Li, Shijie Shang, Zijun Zhai, Meng Wu, Qian Song, Dawei Chen

**Affiliations:** ^1^ Shandong Provincial Key Laboratory of Precision Oncology Shandong Cancer Hospital and Institute Shandong First Medical University and Shandong Academy of Medical Sciences Jinan China; ^2^ Cancer Center, Union Hospital, Tongji Medical College Huazhong University of Science and Technology Wuhan China; ^3^ Cheeloo College of Medicine Shandong University Jinan China

**Keywords:** cancer, gut microbiota and metabolites, radiotherapy

## Abstract

Radiotherapy (RT) is a cornerstone in cancer treatment, but often causes radiation‐induced injury. Accumulating evidence points to the gut microbiota in modulating immune functions and maintaining intestinal integrity to impact RT efficacy. This review examines the current understanding of intestinal flora and their metabolites within the context of RT. We outlined the current research applications in how microbiota‐targeted strategies such as probiotics, prebiotics, dietary interventions, and faecal microbiota transplantation could restore microbial balance, reduce toxicity, and improve patient prognosis. Microbial byproducts such as short‐chain fatty acids, bile acids and tryptophan exhibit protective effects against radiation damage, supporting immune modulation and enhancing tumour radiosensitivity. These microbial products underscore the potential of gut microbiota‐targeted therapies as adjunctive treatments in RT, with implications for reducing toxicity and personalizing cancer care. All these strategies targeting gut microbiota and metabolites potentially aim to develop innovative therapies that boost RT effectiveness while minimizing side effects, and finally revolutionizing personalized cancer treatment.

**Key points:**

- RT alters gut microbiota composition and contributes to intestinal injury and systemic toxicity.
- Gut microbiota regulate mucosal integrity, immune responses and therapeutic outcomes of RT.
- Microbial metabolites, including SCFAs, BAs and tryptophan derivatives, protect against radiation injury and enhance tumour radiosensitivity.
- Microbiota‐targeted interventions (e.g. probiotics, prebiotics, dietary strategies, FMT) show promise for reducing RT‐related toxicity and improving patient prognosis.

## Graphical abstract

Radiotherapy (RT) efficacy and toxicity are influenced by gut microbiota and their metabolites. This review explores how probiotics, prebiotics, and microbial byproducts (e.g., short‐chain fatty acids [SCFAs]) mitigate radiation damage and enhance tumour radiosensitivity. Microbiota‐targeted strategies offer promising adjuncts to RT, potentially personalizing cancer therapy by improving outcomes and reducing side effects.(Source: Created with BioRender.com)

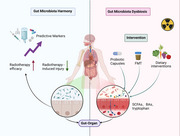

## 1 INTRODUCTION

Despite extensive research spanning decades, cancer continues to be a major global health challenge, characterized by high mortality and morbidity. As the second most common cause of mortality globally, it accounted for almost 10 million fatalities in 2022.^1^, ^2^ The global burden of cancer is exacerbated by the increasing ageing population, lifestyle changes, and environmental factors, which have contributed to a steady rise in the incidence of various cancer types. This growing prevalence points to the critical need for innovative and effective treatments to combat this disease.

**FIGURE 1:**
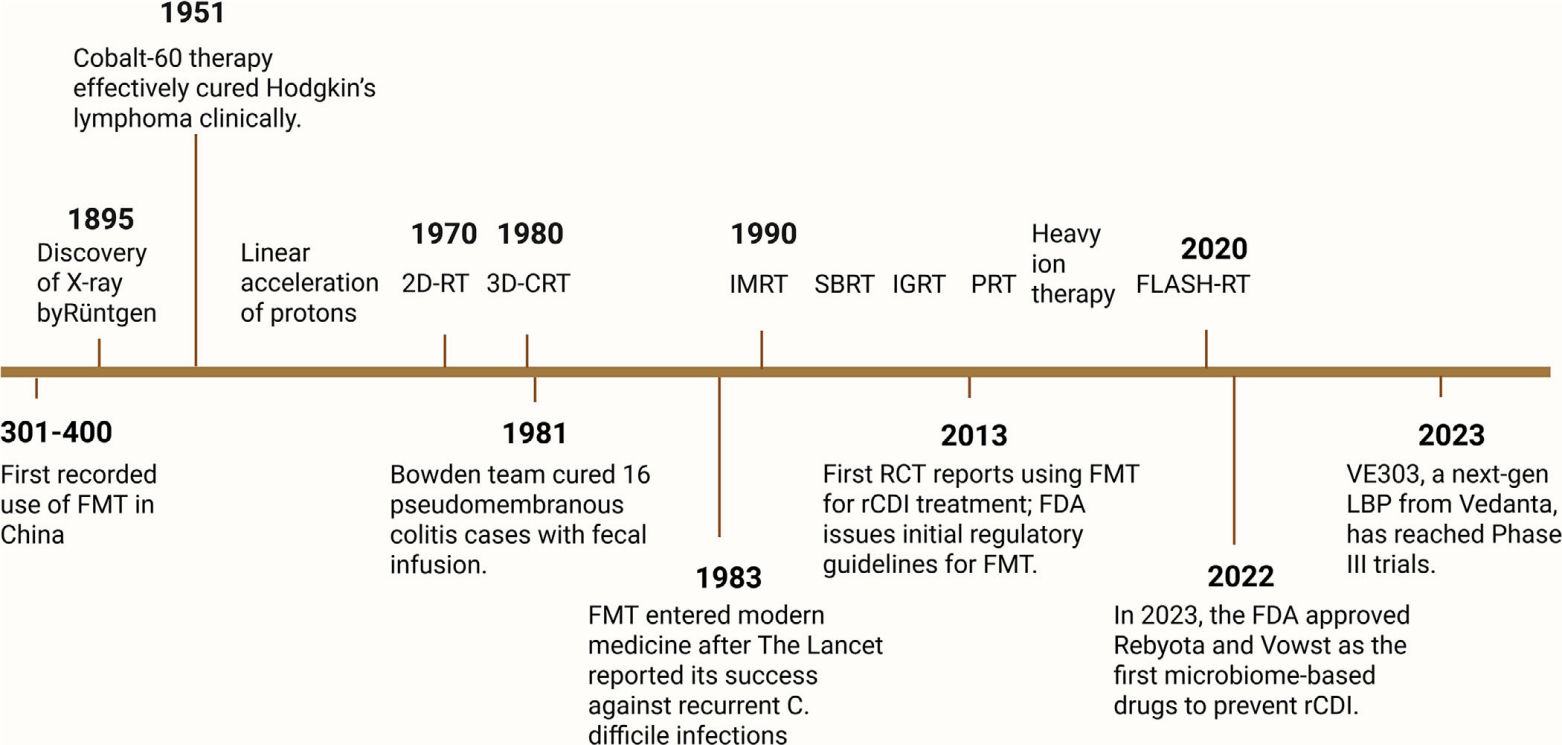
Synergistic development of microbiome‐targeted therapies and radiotherapy techniques. 2D‐RT, two‐dimensional radiotherapy; 3D‐CRT, three‐dimensional conformal radiotherapy; FDA, U.S. Food and Drug Administration; FMT, fecal microbiota transplantation; IGRT, image‐guided radiotherapy; IMRT, intensity‐modulated radiotherapy; LBP, live biotherapeutic product; PRT, proton radiotherapy; rCDI, recurrent *Clostridioides difficile* infection; RCT, randomized controlled trial; SBRT, stereotactic body radiotherapy. *Source*: Created with BioRender.com.

A variety of strategies have been developed for cancer treatment, among which RT remains one of the most commonly utilized methods. As one of the three traditional pillars of cancer therapy, Radiotherapy (RT) primarily aims to improve local disease control by maximizing the cytotoxicity to tumour cell destruction while safeguarding nearby healthy tissues from harm. Over the years, advancements in RT technology have significantly improved the precision and efficacy of RT, reducing side effects and enhancing the therapeutic index.^3^ However, despite these advancements, challenges remain in minimizing radiation‐induced toxicities,^4^ particularly systemic effects such as fatigue, gastrointestinal distress, and immunosuppression, which can severely affect a patient's well‐being. Innovative RT techniques, including FLASH radiotherapy (FLASH‐RT), proton RT, and carbon ion RT, reflect continuous innovations in the field.^5^, ^6^, ^7^ These novel approaches are designed to improve tumour targeting and reduce damage to surrounding healthy tissue, offering promising alternatives to conventional RT (Figure 1). The underlying mechanisms of RT include directly inducing DNA damage in cancer cells through ionizing radiation and indirectly causing DNA damage via reactive oxygen species (ROS).^8^, ^9^ Furthermore, combining RT with immunotherapy has become a viable approach, leveraging the immune system to enhance the tumour's response to radiation.^10^ This combination has demonstrated significant potential in achieving durable responses in various malignancies, particularly in cancers that are otherwise resistant to conventional therapies.

However, despite these advances, radiation injury (RI) remains a significant clinical challenge. RI is often characterized by systemic effects such as fatigue, cachexia, and gastrointestinal symptoms like diarrhoea and rectal bleeding, which severely impact patients' quality of life.^11^, ^12^ While reducing the radiation dose is the standard approach to mitigate RI, it may compromise the therapeutic efficacy of cancer patients.^13^ Therefore, there is a critical need for strategies that can reduce radiation‐induced toxicity while preserving or enhancing the therapeutic effectiveness of RT.

Recent experimental and clinical research have demonstrated the crucial activities of intestinal microbiota and their metabolic derivatives in modulating RT outcomes. The gut microbiota comprises approximately 40 trillion microorganisms inhabiting the human digestive system, predominantly represented by five dominant phyla: *Bacteroidetes, Firmicutes, Proteobacteria, Actinobacteria* and *Fusobacteria*.^14^ This diverse microbial community is essential for gut–epithelial interactions, metabolism regulation, immune system modulation, and inter‐organ communication, including the gut–lung, gut–skin, and gut–brain axes.^15^, ^16^ By engaging in these interactions, intestinal microbiota impact cancer development through the production of metabolites that alter the tumour microenvironment (TME).^17^ Gut microorganisms contribute to cancer incidence and progression through various mechanisms, such as regulating inflammation and immunity, activating signalling pathways, and modulating intestinal metabolism.^18^, ^19^ Notably, the metabolites produced by gut microbiota strongly impact tumour modulation.^20^ These metabolites regulate the TME, influence DNA repair, alter protein epigenetics, and generate ROS, which contribute to DNA damage.^21^, ^22^ Certain bacterial species are closely linked to cancer development.^23^, ^24^ For example, *Helicobacter pylori* promotes the development of gastric cancer and MALT lymphoma.^25^ *Salmonella* induces gallbladder cancer.^26^ In colorectal cancer (CRC), species such as *Fusobacterium nucleatum*, *Bacteroides fragilis* and *Escherichia coli* are implicated in disease progression.^27^, ^28^, ^29^ Moreover, the TcdB toxin from *Clostridium difficile* impacts CRC by regulating the Wnt/β‐catenin pathway and inducing pro‐inflammatory reactions.^30^ Toxin‐secreting *B. fragilis* and *E. coli* producing colibactin drive tumorigenesis through toxin‐mediated mechanisms,^31^ while *Fusobacterium* induces tumour growth by producing tumour‐promoting metabolites.^32^, ^33^, ^34^ Conversely, beneficial bacteria like *Lactobacillus reuteri* exhibit anti‐tumour effects, as demonstrated by reuterin‐induced protein oxidation and suppression of ribosome biogenesis. Another metabolite, indole‐3‐lactic acid (ILA), also demonstrates anti‐tumour properties in human and mouse colorectal cancer models.^35^, ^36^

As a frontline treatment in cancer therapy, RT has shown significant efficacy against various tumours. However, radiation‐induced systemic toxicity and immunosuppression have highlighted important challenges. Interestingly, certain bacterial properties can mitigate these adverse effects. In essence, the microbiome and its metabolites may influence host metabolism and immunity by modulating the TME.^24^ This, in turn, can affect anti‐tumour immunity, ultimately modulating radiosensitivity and impacting the effectiveness of RT.^37^ On the other hand, radiation can upset the natural balance of the intestinal microbiome, resulting in the proliferation of pathogenic bacteria and a reduction in advantageous ones, which can result in complications such as diarrhoea, intestinal wall damage, and inflammation. RT‐induced gut microbiota dysbiosis may further exacerbate radiation‐associated conditions, including radiation enteritis (RE). To counter these effects, interventions like probiotics supplementation or faecal microbiota transfer (FMT) may restore gut microbiota composition, boost immune responses to RT, enhance tumour sensitivity to radiation, and ultimately improve therapeutic efficacy while mitigating radiation‐induced adverse events.^37^

In this review, we explore the critical influence of the intestinal microbiome and its byproducts on the outcomes of RT. The discussion highlights how RT alters the composition and functionality of intestinal flora, resulting in dysbiosis that worsens radiation‐related damage. We also discuss microbiota‐targeted strategies, including probiotics, prebiotics, dietary adjustments, and FMT, emphasizing their ability to rebalance microbial communities, mitigate toxicity, and enhance therapeutic efficacy in RT. Additionally, we discuss the protective and therapeutic roles of microbial metabolites, including short‐chain fatty acids (SCFAs) and bile acids (BAs), in reducing radiation‐induced side effects and improving immune responses in RT. This review synthesizes existing knowledge to offer perspectives on utilizing microbiota‐targeted approaches as non‐invasive predictive biomarkers and adjunctive therapies in RT, with a focus on improving treatment outcomes of cancer patients.

## 2 RADIOTHERAPY MODULATES GUT MICROBIOTA AND THEIR METABOLITES

### 2.1 Radiotherapy reshapes gut microbiota

RT, while effectively targeting cancer cells, profoundly affects the gut microbiota by reshaping its composition and functions (Table 1). The radiation‐induced dysbiosis reduces microbial diversity, alters the quantity of specific advantageous and pathogenic bacteria, and disrupts critical microbial metabolites. These metabolites are essential for modulating TME, preserving the completeness of the protective layer in the gut, and controlling inflammatory responses. Consequently, shifts in intestinal microbiota and their metabolites can either enhance or hinder RT efficacy while modulating radiation‐induced toxicity^17^ (Tables 2 and 3).

**TABLE 1:** Alterations in gut microbiota profiles after radiotherapy.

Model	Radiation/Dose	Biospecimen	Analytical techniques	Alterations in gut microbiota profiles	Reference
Healthy Human	[131I]NaI, [99mTc]NaTcO4, [223Ra]RaCl2	Feces	16S rDNA sequencing	*Firmicutes*↑, *Proteobacteria*↑, *Bacteroidetes*↓, *Actinobacteria*↓	^38^
Animal model (C57BL/6 surviving mice)	8.2–9.2 Gy of TBI	Feces	16S rRNA gene sequencing	*Lachnospiraceae*↑, *Enterococcaceae*↑	^39^
Animal model (C57BL/6 mice)	18 Gy X‐irradiation	Feces	16S rDNA sequencing	*Bacteroidetes*↓, *Firmicutes*↓	^40^
Animal model (C57BL/6 mice)	0–12 Gy of TBI	Feces	16S rRNA gene sequencing	*Phylaproteobacteria*↑*, Bacteroides*↑, *phylum Tenericutes*↓, *genus Roseburia* ↓	^41^
Animal model (C57BL/6 mice)	20 Gy X‐rays Cranial‐irradiation	Feces	16S RNA gene sequencing	*Muribaclum*↑, *Parabacteroides* ↑, *Akkermansia* ↓, *Helicobacter*↓	^42^
Animal model (C57BL/6 mice)	8 Gy X‐ray of TBI	Colon contents	16S RNA gene sequencing	*Proteobacteria*↑ *Bacteroidetes* ↓, *Firmicutes*↓	^43^
Animal model (BALB/c mice)	0.5 Gy of low‐dose Co60 irradiation	Feces	16S RNA gene sequencing	*Clostridium*↑, *Helicobacter*↑, *Bacteroides*↓, *Barnesiella*↓	^44^
Animal model (Chinese rhesus macaques, *Macaca mulatta*)	7.4 Gy cobalt‐60 gamma‐radiation	Feces	16S RNA gene sequencing	*Actinobacillus*↑, *Bacteroides*↑, *Prevotell*a↑, *Veillonell*a↑, *Acinetobacter*↓, *Aerococcus*↓	^44^

**TABLE 2:** Bacterial species/metabolites and mechanisms in radiotherapy.

Type of cancer	Microbial species/Metabolite	Related mechanism	Effect on radiotherapy	Reference
Melanoma or neuroblastoma	CpG ODNs	Activates DCs and B cells via TLR9, enhances antigen presentation and T cell activity	Sensitization	^45^
Colorectal cancer	Escherichia coli (with CD gene)	Converts 5‐FC into 5‐FU, increasing radiation cytotoxicity	Sensitization	^46^
Breast cancer and melanoma	Commensal fungi (*Malassezia* spp)	Promotes carcinogenesis and suppresses immune response via the Dectin‐1 pathway	Resistance	^47^
	Commensal bacteria(*Clostridiales*, *Lactobacillales* and *Burkholderiales)*	Supports anti‐tumour CD8^+^ T cell response	Sensitization	
Colorectal cancer	*Roseburia intestinalis/*butyrate	Enhances radiosensitivity via induction of OR51E1‐RALB‐mediated autophagy, further facilitating tumour cell elimination	Sensitization	^48^
Lung cancer	*Bifidobacterium infantis*	Reduces HIF‐1α expression, enhances DSBs and tumour cell death.	Sensitization	^49^
Colorectal cancer	Butyrate	Enhances radiosensitivity through HDAC inhibition–mediated FOXO3A/p21 activation. Reduces radiosensitivity by suppressing STING‐TBK1‐IRF3.	Dual role	^50^, ^51^
Bladder cancer	Propionate	NA	Sensitization	^52^

Abbreviations: CpG ODNs, cytosine‐phosphate‐guanine oligodeoxynucleotides; DSBs, DNA double‐strand breaks; HDAC, histone deacetylase; NA, not applicable; TLR9, Toll‐like receptor 9.

**TABLE 3:** Microbiome biomarkers and potential applications.

Biomarker category	Specific marker	Predictive direction	Related cancer types	Clinical application	Validation status (study type)	References
Bacterial Taxa	*Fusobacterium nucleatum*	Radiation sensitivity↓	Colorectal cancer	Efficacy prediction and therapeutic target	Preclinical→Translational research	^53^
	*Lachnospiraceae Bifidobacterium*↑	Acute diarrhoea severity↓	Rectal cancer	Toxicity risk stratification	Pilot clinical study	^54^
	*Akkermansia muciniphila↑*	Myelosuppression risk↓	Rectal Cancer	Myeloprotection prediction	Pilot clinical study	^55^
	*Romboutsia↑*	Post‐RT survival time↑	NA	Survival prediction	Mouse model validation	^56^
	SCFA producers (*Clostridium IV*, etc.)↑	Acute/chronic RE symptoms↑	Prostate cancer	Inflammation regulation assessment	Multicenter prospective cohort study	^57^
Microbial Diversity	α‐diversity index (SDI)↓	Acute radiation‐induced diarrhoea risk↑	Gynaecological/Colorectal/Anal cancers	High‐risk patient screening	Multicenter clinical study	^58^
	Chao index↓	Acute/chronic RE risk↑	Prostate cancer	Symptom persistence prediction	Single‐center study ∖	^57^
Metabolites	Butyrate	Acute RE symptoms↑	Prostate cancer	Metabolic intervention target	Multicenter prospective cohort study	^57^
	Fatty acid	Chronic RE symptoms↑	Prostate cancer	Long‐term toxicity management	Multicenter prospective cohort study	^57^

Abbreviations: NA, not applicable; RE, radiation enteritis; SCFA, short‐chain fatty acids; SDI, Shannon diversity index.

#### 2.1.1 Thoracic radiotherapy

Research conducted on mice has demonstrated that localized chest irradiation increases the abundance of *Akkermansia*, *Desulfovibrio*, and *Parasutterella*, while *Rikenella* levels are decreased.^59^ This shift in microbial communities can influence inflammation pathways by altering the production of SCFAs, which are known to exert anti‐inflammatory activities and bolster the resilience of the enteric barrier, ultimately impacting the tumour's responsiveness to radiation. Similarly, M. Thandar et al.^60^ observed that chest irradiation increased *Bacteroidetes* and *Campylobacteria* levels, while reducing *Firmicutes* and *Desulfovibrionaceae*. Treatment with adipose‐derived mesenchymal stem cells (ADSC) reverses these imbalances and restores the normal colonic microbiota composition. Notably, ADSC treatment was shown to enhance the diversity of beneficial bacteria, facilitating a restoration of immune function that had been compromised by the radiation‐induced gut microbiota disruption. Additionally, chest irradiation enhances gut microbiota β‐diversity and significantly alters the overall microbiome composition,^61^ which was also observed in proton therapy.^62^ In patients receiving thoracic RT, the relative abundance of *Actinobacteria* and *Acidobacteria* increases, while *Bacteroidetes* and *Tenericutes* decrease.^38^, ^63^ These changes in microbial populations are likely to modulate the local immune system by adjusting the production of bacterial metabolites, which can either bolster or impair the anti‐tumour immune response. Moreover, Lin et al.^63^ document an increase in *E. coli*, *Cyanobacteria*, *Streptococcus*, *Actinobacteria*, and *Bifidobacterium*, alongside a decline in *Faecalibacterium* and *Bacteroides* levels with the progression of thoracic RT. The shift towards pathogenic bacteria such as *E. coli* and *Streptococcus* may increase inflammation and contribute to RT‐induced toxicities, further complicating treatment efficacy. In a Phase II clinical study conducted with non‐small‐cell lung cancer (NSCLC) patients, thoracic concurrent chemoradiotherapy increased *Bacteroidetes* levels and lowered *Firmicutes* abundance, resulting in a reduced *Firmicutes*/*Bacteroidetes* (F/B) ratio.^64^ These changes in the intestinal flora contributed to the modulation of immune cell activity, which is critical for the effectiveness of RT.

#### 2.1.2 Pelvic radiotherapy

In patients receiving pelvic RT, there are increased *Fusobacterium* levels, along with reductions in *Firmicutes*, *Faecalibacterium* and *Bifidobacterium*.^65^ This disruption is caused by the combined effects of direct radiation and alterations in the intestinal microenvironment. Interestingly, beneficial bacteria like *Lactobacillus* and *Bifidobacterium* can gradually recover from RI over time, leading to a positive shift in the gut microbiota.^66^ Notably, patients whose gut microbiota retains 60% similarity to that of healthy controls during RT are less likely to develop diarrhoea, while those with diarrhoea show only 29% similarity by the end of treatment.^67^ These results indicate that preserving a diverse and balanced microbiome during RT may protect against gastrointestinal side effects of RT. Furthermore, mice that withstand substantial radiation exposure and achieve typical lifespans exhibit significantly enriched bacterial families, including *Lachnospiraceae* and *Enterococcaceae*, which contribute to blood cell regeneration, alleviate gut injury, and aid in recovery from RI.^39^ These bacteria may produce metabolites that promote immune cell proliferation and repair tissue damage, highlighting their potential role in enhancing recovery after RT.

#### 2.1.3 Total body irradiation

In total body irradiation (TBI) models, gut microbiota structure correlates with the level of radiation exposure. For instance, at 4 Gy, *C. innocuum* is predominant; at 8 Gy, *Alistipes*, *Parasutterella* and *Parabacteroides* dominate; while at 12 Gy, taxa such as *E. coli*, *Lachnospiraceae NKRA136* and *Ruminococcaceae UCG014* are most abundant.^41^ These dose‐dependent changes in microbial composition further suggest that varying radiation doses can lead to different microbial communities that may modulate treatment responses and toxicity levels. Additionally, the relative abundance of specific taxa like *Proteobacteria*, *Shigella*, *Xylophilus* and *L. murinus* shows a proportional association with radiation exposure levels, emphasizing a dose‐dependent effect of RT on the structure of the gastrointestinal microbiome.^41^ In another TBI mouse study, Jameus et al.^68^ observed an increase in *Clostridium*, *Verrucomicrobia* and *Bacteroidaceae* in both the colon and stool samples of the RT group. RT‐induced gut microbiota changes also influence metabolic processes in distant organs via the gut–organ axis. For example, in a preclinical study, mice exposed to brain ionizing radiation exhibit significant gut microbiota alterations accompanied by microbial metabolite changes in stool, serum, and the cerebral cortex, indicating that radiation‐induced metabolic effects in distant organs may be mediated by gut micro‐organisms.^42^

#### 2.1.4 FLASH radiotherapy

FLASH‐RT, utilizing exceptionally high dose rates (≥40 Gy/s), has shown remarkable advantages over conventional RT (2 Gy/min). FLASH‐RT has also been reported to increase the balance of *B/F* ratio along with *Prevotella* and *Lactobacillus* levels, while preserving small intestine integrity and mitigating RT‐induced injury.^69^

In conclusion, the intestinal microbiota not only influences the local environment of the gut but also plays a pivotal part in regulating the overall response to RT. By understanding how specific microbial populations and their metabolites influence radiation treatment, we can identify strategies to manipulate the microbiota for optimized RT effectiveness, reduce toxicity and enhance recovery. This evolving research domain underscores the value of microbiota‐based approaches as supportive treatments in RT.

### 2.2 Radiotherapy sways microbial metabolites

Gut microbiota are vital for preserving bodily equilibrium and stability, primarily through the generation of metabolites such as SCFAs, BAs and tryptophan derivatives. RT impacts these microbial metabolites by altering the intestinal microbiota structure, notably reducing the number of bacteria responsible for SCFA production, like *Lachnospiraceae*, *Roseburia* and *Bifidobacterium*. This reduction can disrupt intestinal health and potentially influence radiation‐induced outcomes. In addition to SCFAs, tryptophan‐derived indole compounds, such as indoxyl sulfate and indole‐3‐aldehyde, exhibit radioprotective effects, as demonstrated in mice exposed to radiation.^70^ These compounds can help to mitigate radiation‐induced injury (RII) and facilitate recovery from it. Furthermore, there is a notable shift in bile acid metabolism (BA metabolism), accompanied by reduced secondary bile acids and elevated primary bile acids after total abdominal irradiation (TAI).^71^ Mechanistically, primary bile acids, derived from cholesterol in the liver, are subsequently converted into secondary bile acids by intestinal flora.^72^ Secondary bile acids are essential in radioprotection by activating the membrane receptor GPBAR1 (also known as TGR5), which stimulates intestinal stem cell proliferation and regeneration.^73^ This process highlights the necessity of sustaining a well‐balanced gut microbiota to support recovery and resilience during RT.^74^ Additionally, specific bacterial species can regulate immunity by modulating cytokine synthesis and influencing regulatory T cells (Tregs), which subsequently contribute to the overall recovery process. Maintaining gut microbial homeostasis contributes significantly to successful RT outcomes and fewer adverse effects.

## 3 GUT MICROBIOTA AND THEIR METABOLITES AFFECT THE EFFICACY OF RADIOTHERAPY

### 3.1 Impact of gut microbiota composition on radiotherapy outcomes

The intestinal microbiota and their metabolites are crucial in influencing RT efficacy. Emerging research highlights how the structure and function of the intestinal microbial community can greatly affect radiation response. For example, in colorectal tumours, the gut symbiotic bacterium *Ruminococcus intestinalis* enhances CRC sensitivity to RT by promoting radiation‐induced autophagy through the butyrate/OR51E1/RALB axis. *R. intestinalis* not only improves CRC RT efficacy but also enhances patients’ prognosis, positioning it as a promising radiosensitizer.^48^ This interaction between gut microbiota and RT is bidirectional.^75^ For instance, Sánchez‐Alcoholado et al.^76^ discovered that in CRC patients undergoing neoadjuvant radiochemotherapy (RCT), responders showed a significant enrichment of probiotic bacterium, such as *Bifidobacterium, Ruminococcus, Roseburia* and *Faecalibacterium*. In contrast, nonresponders have an increased abundance of harmful microbes like *F. nucleatum, B. fragilis, E. coli, Parvimonas micra*, and *Klebsiella*, suggesting that gut microbiota may influence RT sensitivity. Apart from CRC, gut microbiome dysbiosis characterized by elevated levels of *Streptococcus* also reduces the efficacy of RT in hepatocellular carcinoma (HCC) by impairing antigen presentation and T cell activities via the cGAS/STING/IFN‐I signalling cascade. Conversely, higher levels of *Clostridiales, Ruminococcaceae* and *Faecalibacterium* are positively correlated with the cytotoxic ability of effector T cells within the TME in HCC.^77^ Beyond bacteria, oncolytic viruses combined with RT hold great potential for cancer therapy. Xu et al.^78^ developed RadioOnco, a novel oncolytic adenovirus that improves systemic delivery, stimulates anti‐tumour immunity, and effectively overcomes RT resistance while preventing metastasis and recurrence, highlighting strong prospects of the virome in RT for clinical translation.

### 3.2 Microbial‐derived metabolites as modulators of radiotherapy response

Microbial metabolites also serve a crucial function in determining RT outcomes. SCFAs, primarily butyrate, propionate, and acetate, are key byproducts secreted by intestinal microbiota through the breakdown of insoluble dietary fibre, resistant starch, and undigested proteins.^79^ Acting as energy sources, SCFAs support mucosal development, improve colonic blood circulation, boost water and electrolyte uptake and maintain intestinal homeostasis and epithelial integrity.^80^ SCFAs are also involved in regulating systemic immune reactions, modulating the equilibrium between pro‐inflammatory and anti‐inflammatory pathways. Specifically, butyrate has been shown to regulate the differentiation of Tregs, promoting anti‐inflammatory responses that help mitigate radiation‐induced inflammation in the TME. Additionally, SCFAs are reported to regulate energy metabolism, improve barrier functionality, alleviate inflammation, and impede tumour growth.^81^ For example, Park et al.^50^, ^82^ demonstrate that butyrate suppresses CRC cell proliferation by regulating genes involved in the cell cycle (e.g., p21, p57 and GADD45) via TOXO3A. As a result, butyrate increases CRC cell sensitivity to radiation and amplifies radiation‐induced cell death. Importantly, butyrate selectively targets cancer cells without inducing the death of normal cells, which suggests that butyrate could improve RT effectiveness and protect normal colon cells, highlighting its potential as a protective adjuvant in RT for CRC.^50^ Beyond the well‐recognized anti‐inflammatory effects in the TME, butyrate has emerged as an important regulator of anti‐tumour immunity with implications for RT efficacy. Increasing evidence indicates that butyrate does more than modulate Treg differentiation; it also shapes effector T cell responses. By promoting cytokine secretion and restoring T‐cell receptor (TCR) signalling, butyrate can overcome inhibitory checkpoints such as PD‐1, thereby reinvigorating exhausted CD8⁺ T cells and enhancing their cytotoxic potential.^83^ In addition, SCFAs have been shown to engage IL‐12–dependent signalling cascades that foster a more active CD8⁺ T cell phenotype,^84^ while also augmenting the performance of engineered immune cells such as CAR‐T cells.^85^ These observations collectively suggest that microbiota‐derived metabolites modulate T cell function at multiple levels, thereby amplifying immune‐mediated tumour control. Such immunostimulatory activities may synergize with radiotherapy by enhancing tumour cell killing and reshaping the immune landscape to support durable anti‐tumour responses.^86^ In addition to CD8⁺ T cells, SCFAs, particularly butyrate, enhance RT efficacy by promoting anti‐tumour immunity through the activation of dendritic cells (DCs) and macrophages, triggering improved antigen presentation and immune system activation.^87^, ^88^ While butyrate‐producing bacteria generally have tumour‐suppressing effects,^89^ butyrate can also exhibit contradictory roles. For example, depletion of butyrate‐producing bacteria by vancomycin enhances anti‐tumour responses to radiation. Butyrate inhibits STING pathway activation in DCs by blocking TBK1 and IRF3 phosphorylation, therefore suppressing type I interferon expression and reducing the tumour‐specific cytotoxic T cell response induced by irradiation. These findings imply that, under certain conditions, butyrate may counteract the anti‐tumour effects of RT.^90^ Thus, vancomycin decreases butyrate‐producing bacteria, enhancing RT sensitivity both at the tumour site and throughout the body via abscopal effects.^91^ In summary, SCFAs exhibit dual roles, acting as both anti‐tumour agents and factors that reduce RT sensitivity by modulating the TME. In addition to SCFAs, other microbial metabolites, including indole‐derived compounds, have demonstrated anti‐tumour properties and potential to enhance RT efficacy.^92^ Teng et al.^93^ found that nucleoside supplementation improves the survival of irradiated cancer cells by promoting efficient DNA damage repair. Additionally, nucleotides synthesized by *B. vulgatus* in the gut diminish the response to RT by enhancing DNA repair capabilities in rectal cancer patients, which indicates that nucleoside levels are correlated with RT outcomes.^93^

**FIGURE 2:**
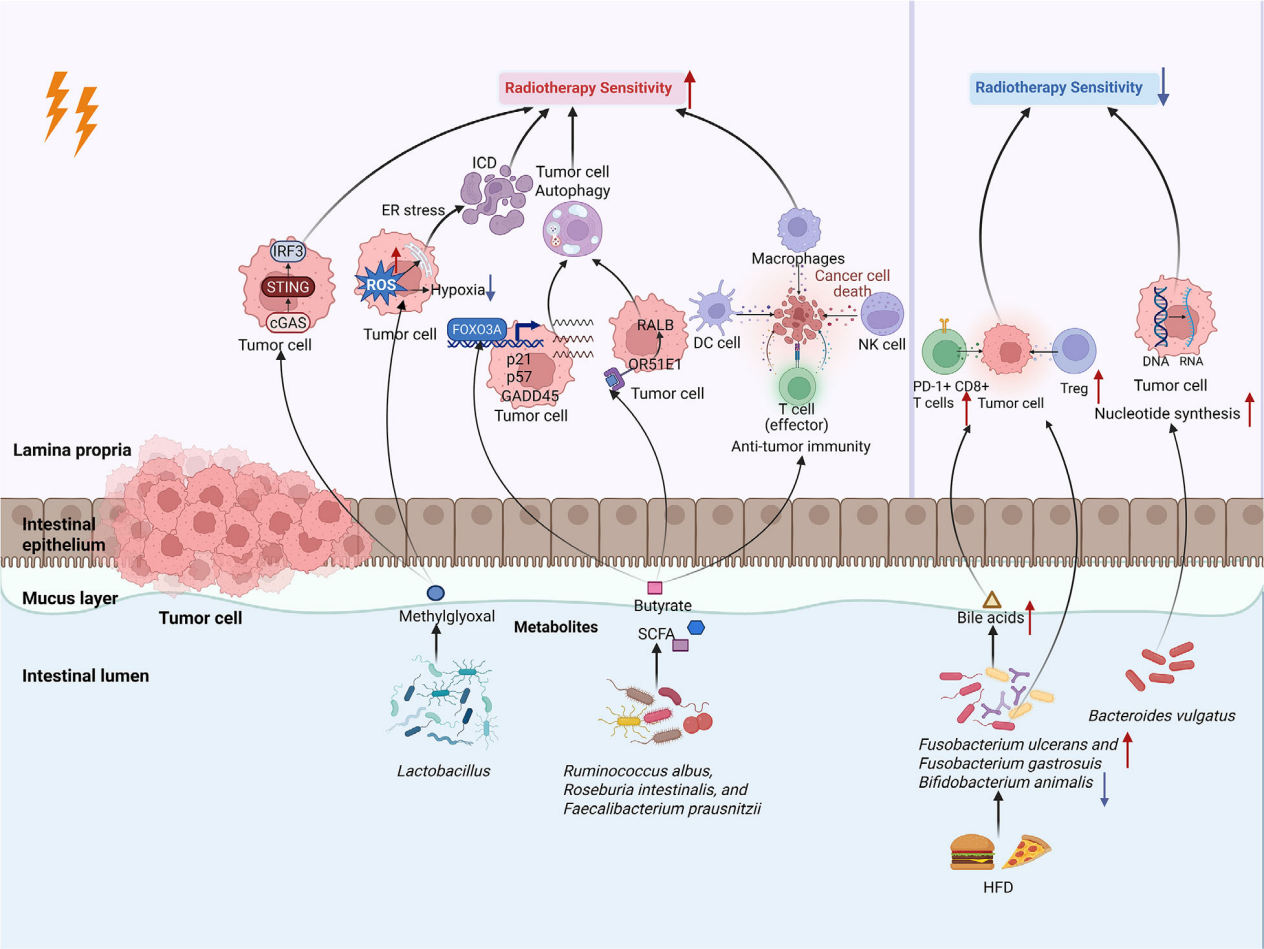
Interactions between gut microbiota, metabolites and radiotherapy in colorectal cancer: modulation of tumour microenvironment and radiotherapy sensitivity. Diagram illustrating the interactions among intestinal microbiota, microbial metabolites, and RT sensitivity in CRC. The gut symbiotic bacterium *Roseburia intestinalis* enhances CRC sensitivity to RT by promoting radiation‐induced autophagy via the butyrate/OR51E1/RALB axis. SCFAs, such as butyrate, regulate p21, p57 and GADD45 via FOXO3A, enhancing CRC cells’ sensitivity to RT while protecting normal tissues and reducing toxicity. Additionally, microbial byproducts such as methylglyoxal act as radiosensitizers by increasing ROS, reducing tumour hypoxia, and activating the cGAS‐STING pathway, ultimately enhancing immune responses and improving treatment outcomes. Conversely, harmful microbial alterations, such as increased nucleotide synthesis by *Bacteroides vulgatus* or dysbiosis induced by an HFD, reduce RT sensitivity by promoting radiation resistance and enhancing DNA repair mechanisms. This bidirectional interaction highlights the pivotal function of the intestinal microbial community in influencing the effectiveness and side effects of RT. CRC, colorectal cancer; HFD, high‐fat diet; ROS, reactive oxygen species; RT, radiotherapy; SCFAs, short‐chain fatty acids. *Source*: Created with BioRender.com.

Beyond SCFAs and nucleosides, Zhou et al.^94^ have identified another microbiota‐derived metabolite, methylglyoxal (MG), as a potential radiosensitizer that increases the effectiveness of RT and radioimmunotherapy in rectal cancer. MG boosts RT sensitivity by raising ROS levels, mitigating tumour hypoxia, activating the cGAS‐STING pathway and ultimately strengthening immune responses.^94^ In contrast, a high‐fat diet induces the increased abundance of *F. ulcerans* and *F. gastrosuis*, and the reduction of *B. animalis*, along with elevated BA levels.^95^ These elements collectively result in the infiltration of Tregs and PD‐1+CD8+ T cells, fostering radiation resistance in rectal cancer.^96^ The interactions between gut microbiota, metabolites and RT in CRC are summarized in Figure 2. Two metabolites from the tryptophan pathway, indole‐3‐acetaldehyde(I3A) and N‐acetyl‐5‐hydroxytryptamine, can also serve as radioprotectors to prevent and alleviate RE by impacting the TME.^97^ In addition to these two metabolites, in a mouse model of HCC, bacterial‐derived C‐di‐AMP interacts with double‐stranded DNA (dsDNA) in irradiated tumour cells, promoting dendritic cell maturation and antigen presentation. This is mainly mediated by activating the cGAS‐STING‐IFN‐β cascade to boost the cytotoxic ability of CD8^+^ T cells.^77^ Another study demonstrates that feeding psyllium with resistant starch enhances tumour radiosensitivity, with increased *Bacteroides* abundance and higher caecal isoferulic acid levels, suggesting that dietary interventions may modulate the microbiota to potentiate RT responses.^98^

### 3.3 Immunomodulatory role of gut microbiota in radiotherapy‐induced immunity

In recent years, the immunoregulatory functions of the intestinal microbiota in cancer therapy have drawn increasing attention.^99^ In the context of RT, accumulating evidence shows that gut microbes not only contribute to maintaining intestinal barrier integrity and metabolic balance but also engage in complex interactions with the immune system, thereby significantly shaping anti‐tumour immune responses following RT.^100^

**FIGURE 3:**
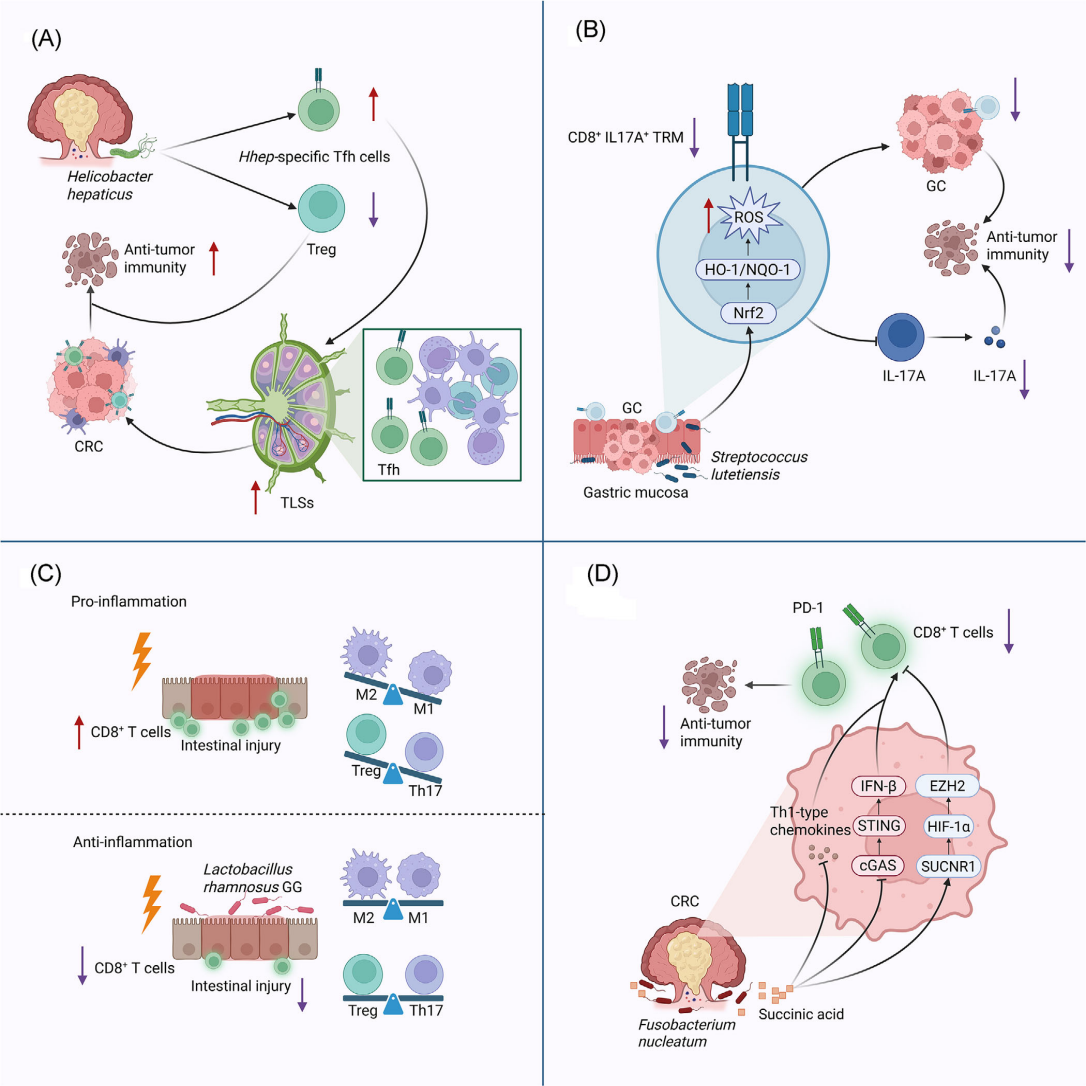
Gut microbiota‐mediated regulation of essential immune cell populations. (A) *Hhep* drives a shift towards higher numbers of CD4⁺ Tfh cells and fewer Tregs. These *Hhep*‐specific Tfh cells facilitate the maturation of tertiary lymphoid structures and strengthen anti‐tumour immunity. (B) *Streptococcus lutetiensis* is enriched within tumour tissues of patients with GC. This bacterium suppresses IL‐17 signalling while elevating ROS production via activation of the Nrf2–HO‐1/NQO‐1 pathway. Consequently, it reduces the population of CD8⁺ IL‐17A⁺ TRM cells, thereby significantly impacting anti‐tumour immunity. (C) By decreasing the presence of pro‐inflammatory M1 macrophages and CD8⁺ T cells, and by rebalancing Th17 and Treg cell populations, *LGG* helps mitigate radiation‐induced damage to the intestine and reestablishes immune homeostasis. (D) In CRC, succinate produced by *F. nucleatum* disrupts the cGAS–STING–IFN‐β signalling pathway and lowers intratumoral Th1 chemokine levels. Simultaneously, it triggers the SUCNR1–HIF‐1α–EZH2 cascade, dampening CD8⁺ T‐cell responses and ultimately reducing the tumour's responsiveness to anti‐PD‐1 monoclonal antibody treatment. GC, gastric cancer; M1, M1 macrophages; M2 macrophages, alternatively activated; Tfh, T follicular helper cells; TLS, tertiary lymphoid structures; Treg, Regulatory T cells; TRM, tissue‐resident memory T cells. *Source*: Created with BioRender.com.

Besides its direct cytotoxic effects on tumour cells, RT can trigger immunogenic cell death, leading to the release of tumour antigens and damage‐associated molecular patterns (DAMPs).^101^ These signals activate various immune cells, including DCs, macrophages, and effector T cells.^102^ Such immune cells are crucial not only for eradicating residual tumour cells but also for remodelling the TME through the secretion of cytokines, chemokines, and the formation of localized immune barriers, further inhibiting tumour recurrence and metastasis.^103^ For example, effector T cells and tissue‐resident memory T cells (Trm) can persist within tumours or mucosal sites and rapidly identify and eliminate residual cancer cells,^104^ while Tregs help suppress excessive inflammation and maintain immune homeostasis.^105^, ^106^ Thus, immune cells are pivotal in sustaining RT efficacy, shaping the microenvironment, and influencing long‐term outcomes (Figure 3).

Importantly, gut microbes can affect the function of these immune cells through multiple mechanisms, thereby indirectly influencing RT effectiveness. On one hand, microbial antigens and metabolites exert effects on the host immune system to modulate immune cell differentiation and activation. For instance, Overacre‐Delgoffe et al.^107^ demonstrated that transferring *Hhep*‐specific CD4+ T cells into Tfh‐deficient mice promoted the formation of tertiary lymphoid structures (TLS), thereby restoring anti‐tumour immunity. As immune hubs within the TME, TLS not only activate T cells but also enhance anti‐tumour effects, a process also modulated by RT.^108^

On the other hand, certain bacterial species or their metabolites can influence immune cell infiltration and function by altering cytokine networks. For example, *Streptococcus anginosus* activates the Nrf2–HO‐1/NQO‐1 pathway, elevating ROS levels and consequently reducing the infiltration of CD8⁺IL‐17A⁺ Trm cells and lowering IL‐17A expression, thus promoting gastric cancer progression.^109^ Moreover, *L. rhamnosus GG* (*LGG*) has been shown to reduce infiltration of pro‐inflammatory M1 macrophages and CD8+ T cells after RT while restoring the local balance between Th17 and Treg cells, underscoring the dual regulatory effects of gut microbiota on immune cell quantity and functionality.^43^

Notably, immune cells are not limited to acting locally within tumours but are also key players in mediating the abscopal effect induced by RT. Low‐dose radiotherapy (ILDR), particularly in the range of 1–3 Gy, can enhance CD8+ T cell activation while preventing their exhaustion by modulating host metabolism and interactions with the gut microbiota.^110^ Activated CD8+ T cells and DCs can not only destroy tumour cells directly but also secrete diverse cytokines and chemokines, further reshaping the TME, promoting immune cell infiltration, and inhibiting tumour progression and metastasis.^111^

Further research has revealed close metabolic and immunological interactions between ILDR and the gut flora.^110^ ILDR can alter cholesterol and bile acid (BA) metabolism, leading to cholesterol accumulation within DCs, which enhances their capacity for antigen processing and presentation, thereby strengthening T‐cell activation.^112^ Specific bacterial strains, such as *Christensenella minuta*, have been found to significantly augment the anti‐tumour effects of ILDR combined with PD‐L1 blockade.^110^ This bacterium facilitates the migration of PD‐L1‐high DCs from the gut to tumour‐draining lymph nodes and, by expressing high levels of bile salt hydrolase, promotes fatty acid oxidation while suppressing triglyceride synthesis and hepatic fatty acid production, contributing to a metabolic environment favourable for anti‐tumour immunity.^110^, ^113^

Additionally, gut microbial metabolites like SCFAs and indole derivatives serve as important molecular mediators in microbiota‐immune interactions.^87^ These compounds can influence the differentiation, distribution, and function of immune cells, including Trm and other T cell subsets, thereby potentially enhancing or diminishing the anti‐tumour efficacy of RT.^114^ Moreover, the tryptophan metabolite I3A, produced by *Lactobacillus* within the tumour microenvironment, has been shown to enhance IFN‐γ production via CD8⁺ T cell‐specific activation of the AhR signalling pathway. This mechanism may reshape the TME and subsequently influence the therapeutic efficacy of RT.^115^, ^116^

Overall, intestinal microbiota orchestrates multilayered and diverse mechanisms that modulate immune cell activation, migration, and functionality. In turn, immune cells reshape the tumour microenvironment, regulate inflammation, and influence immune infiltration, collectively determining the therapeutic outcomes of RT and the occurrence of systemic (abscopal) effects. Elucidating the complex crosstalk among the microbiome, the immune system, and RT holds promise for optimizing RT strategies, improving therapeutic sensitivity and reducing side effects.

## 4 GUT MICROBIOTA AND THEIR METABOLITES IN RADIATION INJURY

### 4.1 Radiation‐induced gut injury

The intestines, as the second most radiation‐sensitive organ, are especially vulnerable during RT for abdominal or pelvic malignancies. Inevitable radiation exposure damages the small intestine, colon and rectum, with nearly half of patients undergoing pelvic or abdominal RT developing gastrointestinal mucositis.^117^ Moreover, RT‐induced dysbiosis further exacerbates intestinal injury, underscoring the intricate and close interplay between RT and gut health.

#### 4.1.1 Types and mechanisms of radiation‐induced intestinal injury

Radiation‐induced intestinal injury (RIII) can be classified into two types: acute and chronic. Acute RIII is typically characterized by immediate damage to the bowel's mucosal layer, leading to symptoms such as diarrhoea, which can occur anywhere from 2 to 12 weeks after exposure. In contrast, chronic RIII can emerge months or even years after exposure, with long‐term consequences such as intestinal perforation, blockage, persistent diarrhoea, and nutrient malabsorption.^118^, ^119^ Despite advancements in RT techniques, RE remains a significant obstacle in improving patients' survival outcomes.

The processes underlying RII damage (RIID) involve the disruption of multiple protective barriers within the intestines: the physical, chemical, and immune barriers.^120^ RT directly damages the DNA of intestinal epithelial cells (IEC), particularly the crypt stem cells, which are essential for regenerating the intestinal lining. This damage leads to cell apoptosis, curbing the amount of stem cells and weakening the physical barrier of the intestine.^121^ In addition to damaging IEC, radiation also harms endothelial cells, leading to increased levels of ROS, which cause delayed injury that affects the intestines over a longer period.^121^ The chemical barrier is compromised due to reduced mucus secretion, which increases intestinal permeability and triggers intestinal inflammation.^122^ The immune barrier, responsible for the regulation of immune cells, can also be impaired by radiation. Disruption of this barrier can lead to autoimmune diseases, chronic inflammation, or weakened immune responses, making the body more susceptible to infections and malignancies.^123^

#### 4.1.2 Changes in gut microbiota composition during RIII

An increasing number of studies underscores the shielding function of the gut's microbial community as mitigating radiation‐induced damage, largely by promoting epithelial regeneration and supporting the repair of intestinal tissues.^124^ In patients with RE, *Serratia* and *Coprococcus* levels are elevated, while *Bacteroides* levels significantly decrease, accompanied by a marked reduction in α‐diversity. Alterations in gut microbiota are strongly associated with RE severity, as patients with grade 3 RE exhibit the lowest α‐diversity and the highest β‐diversity.^125^ Moreover, improved survival outcomes have been linked to higher proportions of certain gut microbes, such as *Lactobacillus, Roseburia* and *Akkermansia*, in patients receiving whole‐body irradiation.^126^ Conversely, other studies have shown that certain bacterial genera, including *Fusobacterium, Enterococcus, Escherichia* and *Pseudomonas*, tend to exacerbate radiation‐induced damage, whereas species like *C. sporogenes, B. pseudolongum* and *L. acidophilus* are associated with mitigating the damage.^127^ These compositional shifts underscore the pivotal role of gut microbiota in modulating the severity and progression of RIII.

#### 4.1.3 Protective interventions targeting gut microbiota in RIII

Numerous studies have demonstrated the protective potential of probiotics against RIID. In irradiated mice, administering a complex probiotic blend consisting of *Lactobacillus* and *Bifidobacterium* protects IEC and promotes the proliferation of crypt cells.^128^ This probiotic supplementation also contributes to restoring the diversity of intestinal bacterial species and reduces the risk of acute RIII and gut dysbiosis.^41^ Additionally, another probiotic consortium (*L. plantarum, B. longum* and *L. paracasei*) exhibits therapeutic effect in improving survival rates and alleviating RIII in mice by modulating the composition of intestinal microorganisms, in comparison to a single probiotic strain.^129^ Notably, the spore layers (SGs) of *Bacillus* have demonstrated radioprotective properties by scavenging ROS, reducing inflammation, restoring intestinal flora balance, and promoting *Lactobacillus* growth.^130^ SGs alleviate RIID, inhibit apoptosis in IEC, and enhance weight recovery and survival rates in mice exposed to whole‐abdominal X‐ray irradiation.^130^ Furthermore, oral administration of *E. coli‐*producing IL‐22 is reported to enhance outcomes for patients receiving RT with gastrointestinal syndrome.^126^ Similarly, the *B. fragilis* strain ZY‐312 offers protection against RIID by facilitating IEC proliferation, stem cell renewal, mucus production and maintaining tight junction integrity via the STAT3 signalling pathway.^131^ In addition, *LGG* demonstrates significant potential as a radioprotective agent by activating a cascade reaction via lipoteichoic acid (LTA) to protect intestinal epithelial stem cells,^132^ modulating the cGAS/STING pathway to reduce radiation‐induced oxidative stress.^43^, ^133^ Furthermore, *LGG* enhances the abundance of *Lactobacillus* and reshapes the gut microbiota profile in irradiated mice, highlighting its promising function in reducing radiation‐induced harm and improving intestinal health. Collectively, these findings suggest that *LGG* could become a valuable tool for protecting against radiation damage and enhancing therapeutic outcomes.^43^

In addition to probiotics, certain microorganisms and their byproducts have a substantial impact on counteracting the side effects of RT. SCFAs, such as butyrate, are particularly important for mucosal repair after RT. They help reduce intestinal inflammation, maintain barrier function, and support tissue regeneration. For instance, butyrate can alleviate gastrointestinal damage induced by dietary polysorbate 80 after RT.^134^ Dietary adjustments, such as a fibre‐rich diet, can stimulate the proliferation of fibre‐fermenting bacteria to boost SCFA production.^135^ Dietary fibre, primarily functioning as a prebiotic, is not digested or absorbed but processed by bacteria in the gastrointestinal tract, resulting in the generation of SCFAs.^136^ Inulin promotes the multiplication of SCFA‐producing bacteria in the intestine to help alleviate radiation‐induced colonic fibrosis.^137^ Additionally, CK and colleagues demonstrate that a fibre‐rich diet enhanced tumour control during RT by modulating the intestinal flora, enhancing immune reactions, and reducing radiation‐induced gastrointestinal toxicity in a bladder cancer mouse model.^138^ The impact of dietary interventions extends beyond just fibre. For example, konjac glucomannan (KGM), a type of prebiotic, modulates the gut microbiota and supports the stability within the intestinal environment by increasing the abundance of beneficial bacteria and promoting SCFA production.^139^ KGM also serves as a potential radioprotective agent with properties of promoting recovery of the hematopoietic system and suppressing apoptosis in irradiated human IEC.^139^ Plant polysaccharides, such as buckwheat *Fagopyrum tataricum* polysaccharides and *F. esculentum* polysaccharides, possess prebiotic properties that regulate gut microbiota and cellular immunity by enhancing the population of bacteria that generate SCFAs.^140^ A key amino acid, tryptophan, is vital for numerous physiological functions, including protein synthesis and neurotransmitter production.^141^ Additionally, tryptophan metabolism has been implicated in regulating immune reactions and the protection of tissues from various stressors, including radiation. The metabolites derived from the tryptophan metabolic pathway, such as I3A and kynurenine, have been shown to significantly enhance radiation protection by mitigating oxidative stress and modulating immune responses.^39^ Additionally, tryptophan derivative urolithin A shows potential in alleviating RIID.^135^ I3A also reduces radiation‐induced gut injury by activating the AhR/IL10/Wnt3 pathway to promote IEC proliferation and differentiation.^142^ Moreover, Indole‐3‐propionic acid (IPA), derived from gut microbiota, exhibits radioprotective effects by preserving acyl‐CoA binding protein, reducing inflammation, and mitigating damage to the hematopoietic and gastrointestinal systems. Furthermore, IPA provides these protective benefits without promoting tumour growth, highlighting its potential as an effective radioprotective agent.^143^ Beyond SCFAs and tryptophan derivatives, the disruption of BA metabolism is another key factor in radiation‐induced intestinal damage. Guo et al.^71^ found that RIID is considerably linked to disruptions in BA metabolism. Specifically, lithocholic acid treatment offers protection against TAI‐induced intestinal damage in mice by upregulating the expression of TGR5 and YAP1 in intestinal crypts. FMT has also demonstrated protective effects against RE by modulating tryptophan metabolism, particularly through *Lachnospiraceae* and I3A.^97^

These findings underscore the intricate and interrelated role of the intestinal microbiome and its metabolites in managing RII, highlighting the significance of manipulating the intestinal flora and its metabolic processes to enhance RT results.

#### 4.1.4 Other modulating factors in RIII

The composition of gut bacteria after irradiation is also influenced by circadian rhythms, further complicating the effects of RT. Studies have shown that mice exposed to disrupted light cycles or abnormal circadian rhythms exhibit a reduction in gut bacterial diversity, which is associated with decreased resistance to radiation. This highlights the importance of maintaining a stable circadian rhythm, as any disturbances may affect the intestinal microbiota's ability to modulate radiation sensitivity, potentially impacting therapeutic outcomes and elevating the chances of radiation‐induced damage.^144^ In addition, traditional medicine‐derived natural compounds like Vanillin derivative VND3207 and Ginsenoside Rg3 (GRg3) are also reported to have promising therapeutic potentials in mitigating radiation‐induced damage. Vanillin derivative VND3207 is a natural compound with antioxidant, antimutagenic, and DNA repair properties. VND3207 treatment reverses gut microbiota alterations induced by traumatic brain injury and markedly increases the survival of mice exposed to lethal radiation.^145^ GRg3 regulates the gut microbiome and mitigates acute radiation‐induced proctitis by suppressing the TLR4/MyD88/NF‐κB signalling pathway, which decreases the expression of inflammation‐promoting factors.^146^ Gender differences are also reported to significantly affect radiation‐induced toxicity. Male rats experience more severe acute, subacute, and chronic lung toxicity, while female rats exhibit lower toxicity, possibly due to protective changes in gene expression following radiation in female rats.^147^ Additionally, gender differences are observed in radiation‐induced cardiac toxicity, with lung radiation dose playing a key role in heart function impairment.^148^ These findings highlight important factors for future studies on alleviating radiation‐induced toxicity.^149^ These findings suggest that gender should be considered when designing radiation therapy strategies, as personalized approaches may be needed to address these differences and optimize therapeutic outcomes.^150^ Additionally, caloric restriction has been shown to reduce radiation‐induced damage, and its effects may vary depending on gender, further emphasizing the need for tailored treatment plans.^151^

RE remains a multifactorial challenge, requiring a multifaceted therapeutic approach. Understanding the function of the intestinal microbial community and harnessing its metabolites, probiotics, dietary adjustments, and natural compounds provides a promising strategy to mitigate radiation‐induced toxicity, enhance the body's resilience and improve overall therapeutic outcomes. Moreover, incorporating factors such as circadian rhythms and gender differences into future research will be crucial in developing personalized RT strategies that maximize efficacy while minimizing side effects. With continued research, there is great potential to optimize radiation treatment plans and boost the living standards for individuals with cancer who are in RT treatment.

### 4.2 Radiation‐induced extracolonic injuries

The gut–organ axis describes how the intestinal microbiome, via its metabolites, regulates the health and functionality of various organ systems in the body.^152^ This intricate relationship not only illuminates the central impact of the microbiome in maintaining local gut health but also in regulating systemic processes, including the functionality of organs such as the skin, brain, and oral cavity, which can all be impacted by RT. Understanding the complexity of the gut–organ axis offers insights into novel therapeutic strategies that can mitigate radiation‐induced damage to distant organs, enhancing overall patient outcomes during RT.

#### 4.2.1 Radiation‐induced skin damage

Radiation‐induced skin damage is a frequent and debilitating side effect, particularly for patients undergoing RT for breast, lung, and colorectal cancers. These conditions often lead to severe skin dryness, irritation, and even ulceration, which drastically diminishes patients’ quality of life, causing discomfort and increasing the risk of infection at the treatment site.^153^, ^154^ In addition to the direct physical effects, radiation‐induced skin damage can result in emotional distress, as patients may feel self‐conscious about visible changes to their appearance. The use of probiotics and postbiotics has been reported to mitigate radiation‐induced skin damage by promoting skin regeneration and reducing inflammation.^155^ Microbiota‐derived SCFAs can enhance skin barrier function by modulating epidermal mitochondrial metabolism and boosting keratinocyte metabolism through gut–skin axis.^156^, ^157^ These SCFAs contribute to skin tissue repair by promoting cell proliferation and differentiation, thereby supporting the skin's ability to recover more effectively from radiation exposure. Additionally, SCFAs may also regulate the immune response, helping to prevent excessive inflammation and promoting a balanced healing process.

#### 4.2.2 Radiation‐induced brain injury

Radiation‐induced brain injury (RIBI) potentially leads to cognitive impairment, memory loss and neuroinflammation, which significantly augment the life conditions for cancer patients enduring RT.^158^ The connection between the enteric system and the brain is mediated by diverse microbiota and their metabolites in the gastrointestinal system, which act as critical mediators of brain function and behaviour.^159^ A potential prebiotic called *Lycium barbarum* polysaccharides (LBP) has been reported to alleviate emotional distress and cognitive dysfunction by downregulating the inflammatory cytokine TNF‐α level. This effect helps to alleviate RIBI.^160^ LBP can also reduce the proportion of *Clostridium* and *Burkholderia* while enhancing that of *Lactobacillus*, effectively eliminating radiation‐induced gut microbiota dysbiosis.^160^ Furthermore, quercetin inclusion complex gels have been reported to improve spontaneous activity, short‐term memory, and stress levels through modulation of the microbiota–gut–brain axis. This is achieved by downregulating *Firmicutes* and upregulating *Bacteroidetes*, which influence gut–brain communication and promote cognitive health.^161^ *L. reuteri* microcapsules significantly improve mouse behaviour by regulating the gut microenvironment.^162^ They restore intestinal microbial diversity by promoting beneficial bacteria and suppressing harmful ones, which further lowers inflammatory factors to alleviate neuroinflammation caused by RIBI. This regulation enhances cognitive function, reduces anxiety, and improves memory, highlighting its protective potential via the gut–brain axis.^162^ As well, the application of the probiotic formula can reduce the amount of apoptotic cells and alleviate neuroinflammation in the hippocampal region induced by radiation.^163^ Collectively, these interventions show promise for alleviating RIBI.

#### 4.2.3 Radiation‐induced oral mucositis

Radiation‐induced oral mucositis (OM) is one of the most common oral side effects of head and neck RT, occurring in 80–100% of patients treated for head and neck cancer.^164^ This condition leads to painful ulcers, difficulty in swallowing, and an increased risk of infection, which severely impacts the patient's ability to eat and speak, thus reducing their quality of life. Research by Xiao et al.^165^ revealed that *Streptococci* originating from the oral cavity can migrate to the intestine, emphasizing the interplay between the gut and oral microbiota. Interestingly, antibiotic‐induced depletion of gut microbiota was found to lower interleukin‐6, interleukin‐1β and toll‐like receptor 4 expression in tongue tissue, leading to a reduction in tongue mucosal ulcers and faster recovery from OM.^166^These findings emphasize the metabolic interconnection between the oral and intestinal microbiota, suggesting that restoring microbial balance through probiotics may improve oral homeostasis and exert a notable influence in the management of OM during RT. Probiotic interventions may support recovery from OM by reducing inflammation and promoting tissue repair.^167^

#### 4.2.4 Radiation‐induced lung injury

Radiation‐induced lung injury (RILI) represents a significant dose‐limiting toxicity that limits dosage in chest RT, particularly in lymphoma, breast cancer and lung cancer treatment. RILI can progress to chronic pulmonary fibrosis and potentially lead to respiratory failure and even death.^168^ As the severity of RILI increases, it can seriously impact the patient's overall life quality, requiring adjustments in treatment plans to prevent long‐term lung damage. In a murine RILI model, the combined supplementation of SCFAs significantly reduced the incidence of RILI, suggesting a potential therapeutic role for SCFAs in mitigating RILI.^169^ Moreover, gut microbiota‐derived PGF2α can activate the FP/MAPK/NF‐κB pathway in lung cells to provide a protective effect against radiation‐induced damage by modulating inflammatory responses and tissue repair mechanisms.^59^, ^61^ Cryptotanshinone has been found to alleviate lung inflammation and fibrosis by increasing beneficial genera such as *Enterobacter* and *Akkermansia* and regulating BA metabolites in mice with radiation‐related lung fibrosis.^170^ This regulation helps to restore the gut microbiota's balance and promote lung tissue repair. Furthermore, ImP, a metabolite of L‐histidine derived from the gut microbiome, has demonstrated the ability to mitigate radiation toxicity in both the lungs and heart. ImP not only protects these vital organs but also helps reshape the gut microbiome after RT, promoting overall recovery and resilience against radiation‐induced damage.^171^

#### 4.2.5 Radiation‐induced liver injury

Radiation‐induced liver injury is a common complication following RT for hepatobiliary malignancies and liver metastases, and it can severely impact liver function and overall health.^172^ The liver is particularly vulnerable to signals from the gut due to its unique susceptibility to bacterial products and environmental toxins, which are translocated through the gut–liver axis.^95^, ^173^ This axis plays a crucial role in modulating liver functions, as changes in the intestinal microbiota can significantly influence liver signalling pathways, immune responses, and metabolic processes.^174^, ^175^ For instance, a notable increase in *Burkholderiales* abundance during gastrointestinal microbiome dysbiosis has been strongly associated with compromised gut barrier function and bacterial migration to the liver, and improved inflammatory responses.^176^ Furthermore, radiation‐induced dysbiosis disrupts critical metabolic processes, including one‐carbon metabolism and amino acid homeostasis, leading to increased oxidative stress and metabolic dysfunction in the liver.^176^ These disruptions contribute to the progression of liver damage, making it more challenging for the liver to recover from radiation. Modulating intestinal flora composition shows potential for reducing radiation‐induced liver injury and supporting hepatic repair.^177^

#### 4.2.6 Radiation‐induced hematopoietic system damage

Another radiation‐induced injury is in the hematopoietic system. Bone marrow (BM) exhibits high sensitivity to ionizing radiation. Radiation‐induced hematopoietic dysfunction is mainly caused by damage to hematopoietic stem cells (HSCs) in BM. As HSCs are critical for the production of blood cells, their impairment leads to severe consequences, including anaemia, thrombocytopenia, and leukopenia. Therefore, the remodelling of the BM microenvironment is the key factor for HSC regeneration, followed by RT.^178^ Another study demonstrated that FMT from young mice restores the differentiation potential of lymphoid, rejuvenates aged HSCs, and enhances hematopoietic reconstitution, suggesting that gut‐derived factors play an essential role in supporting HSC recovery.^179^ Additionally, this research found that bacteria from the *Lachnospiraceae* family and tryptophan‐related metabolites effectively promote hematopoietic recovery, as well as the rejuvenation of aged HSCs, potentially offering a new therapeutic pathway for enhancing BM function.^179^ Guan et al.^180^ further revealed that I3A therapy significantly alleviates radiation‐induced hematopoietic damage by expediting blood cell recovery, facilitating BM reconstruction, and improving the functional restoration of hematopoietic stem/progenitor cells (HSPCs). I3A therapy also reduces ROS production, which contributes to its ability to inhibit HSPC apoptosis and enhance the survival of these critical cells. The underlying mechanism involves promoting the quiescence of HSPCs and increasing their resistance to radiation‐induced damage, thereby preserving the regenerative capacity of the hematopoietic system.^180^ Notably, the role of the gut–BM axis has garnered increasing attention over the past few years. Targeting gut‐derived factors offers a promising avenue for mitigating radiation‐induced hematopoietic injury.^181^

**FIGURE 4:**
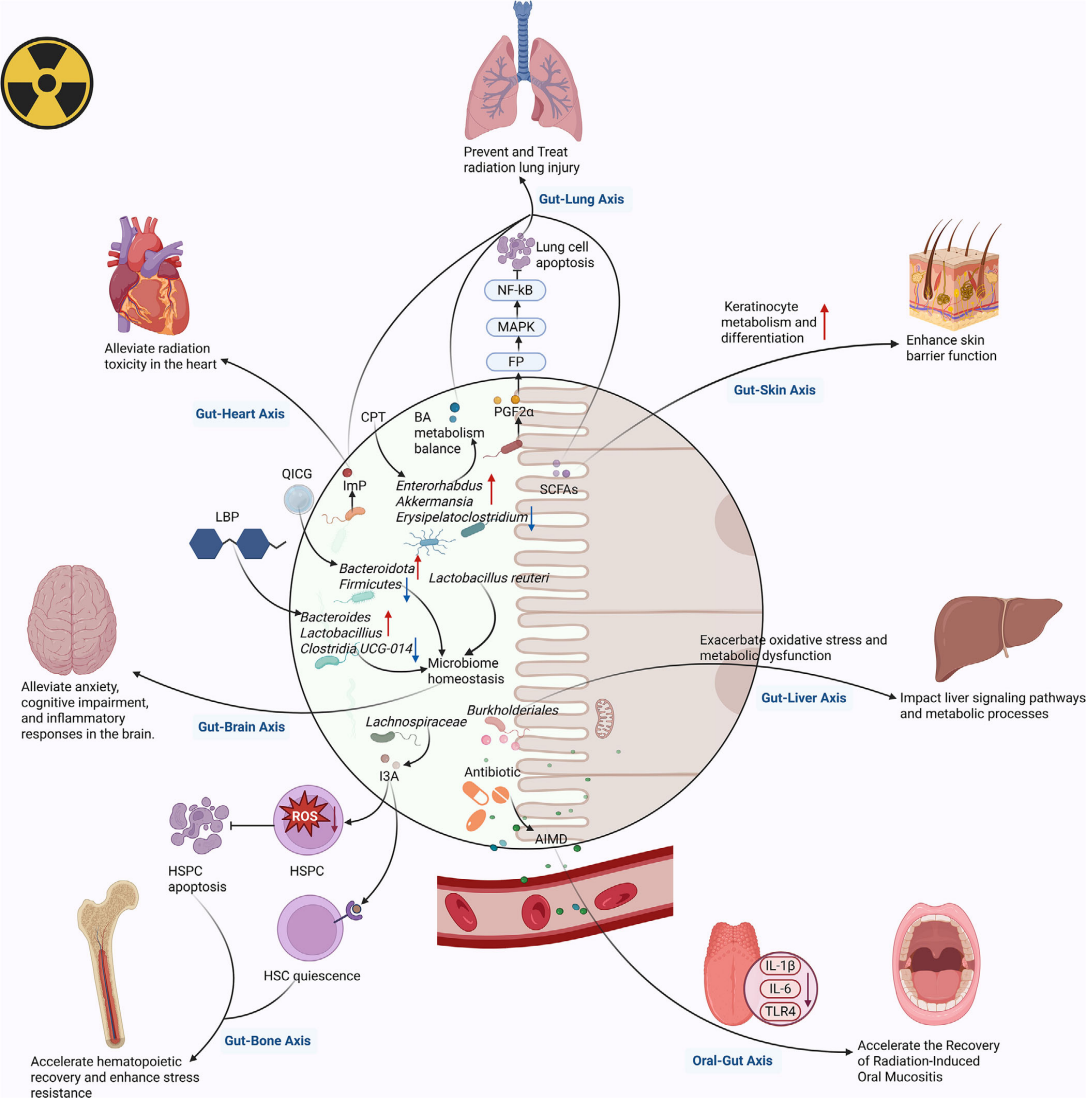
Radio‐protective effects of the gut–organ axis: modulating gut microbiota and their metabolites to mitigate radiation‐induced damage across multiple organ systems. AIMD, antibiotic‐induced‐microbiota depletion; BA metabolism balance, bile acid metabolism balance; CPT, cryptotanshinone; HSCs, hematopoietic stem cells; HSPCs, hematopoietic stem/progenitor cells; I3A, Indole‐3‐acetaldehyde; LBP, *Lycium barbarum* polysaccharides; QICG, quercetin inclusion complex gels; SCFA, short‐chain fatty acids; TLR4, Toll‐like receptor 4. *Source*: Created with BioRender.com.

The gut–organ axis exemplifies the intestinal microbiota's systemic influence, revealing novel insights into mitigating RT‐induced damage across various organs. By leveraging microbiota and their metabolites, future therapies could offer personalized and multi‐targeted interventions, enhancing RT's effectiveness and working to minimize its negative repercussions. Further research into this axis promises to redefine approaches to radiation damage management, bridging the gap between gut health and systemic recovery (Figure 4).

## 5 GUT MICROBIOTA AS PREDICTIVE MARKERS FOR RADIOTHERAPY EFFICACY

**FIGURE 5:**
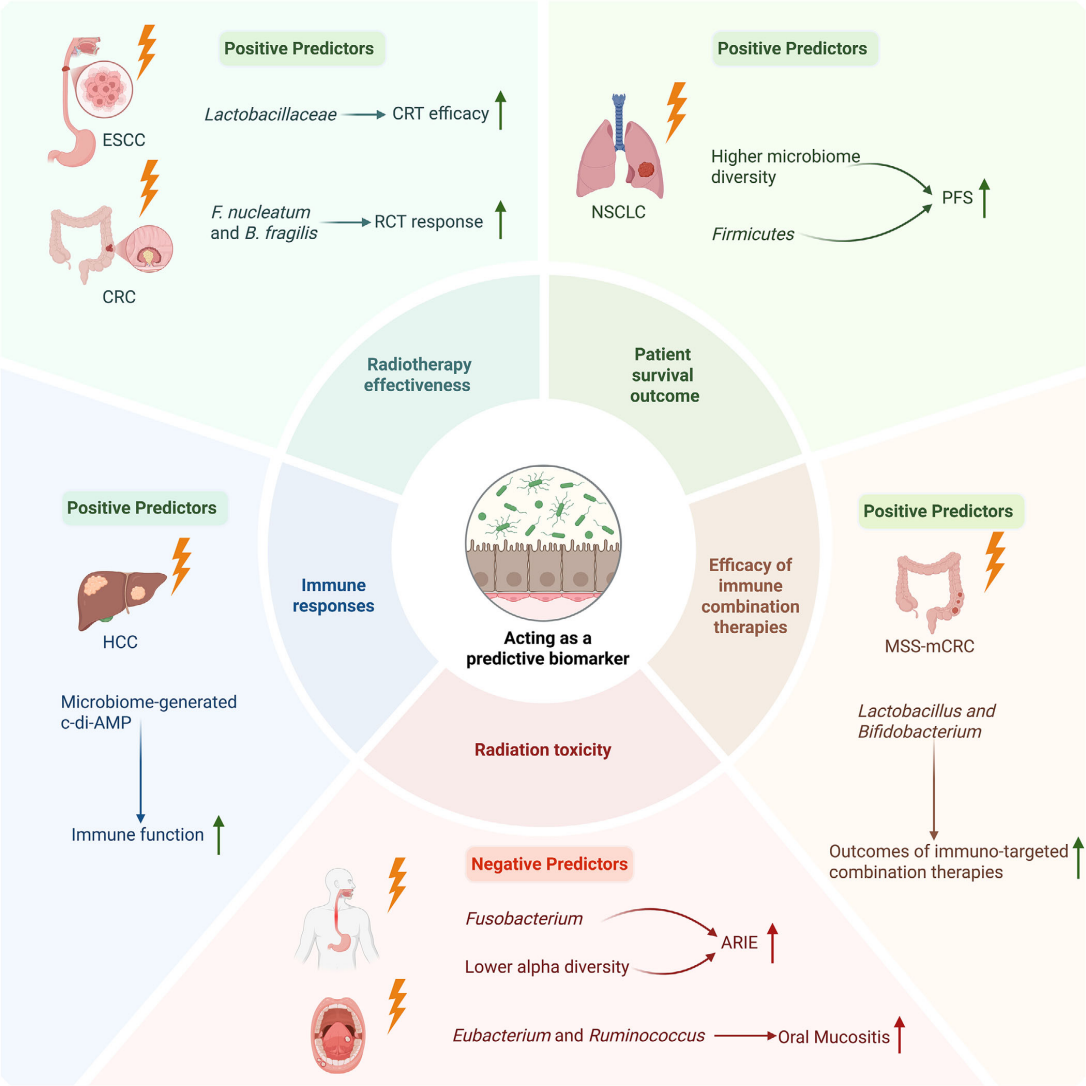
Gut microbiota as predictive biomarkers for radiotherapy outcomes: key microbial taxa and microbiome‐derived factors linked to divergent clinical responses in patients with various cancers. ARIE, acute radiation‐induced esophagitis; *B. fragilis, Bacteroides fragilis*; C‐di‐AMP, cyclic‐di‐AMP; CRC, colorectal cancer; CRT, chemoradiotherapy; ESCC, oesophageal squamous cell carcinoma; F. nucleatum, Fusobacterium nucleatum; MSS‐mCRC, microsatellite stable metastatic colorectal cancer; NSCLC, non‐small‐cell lung cancer; PFS, progression‐free survival; RCT, radiochemotherapy. *Source*: Created with BioRender.com.

Predicting RT efficacy and radiation‐induced injury remains a considerable challenge because of the absence of reliable tools or effective preventive strategies. Amifostine is currently the sole drug authorized by the U.S. Food and Drug Administration (FDA) to prevent radiation toxicity.^182^ However, recent research highlights the growing potential of intestinal flora as a tool to predict RT outcomes and as a target for therapeutic intervention (Figure 5). The gut microbiome's composition and diversity are gaining growing acknowledgement for their part in influencing the effectiveness of RT and patient survival across various cancer types. By understanding and leveraging the microbiome's impact, researchers are working towards more personalized and targeted radiation treatments.

Specific intestinal flora has a strong correlation with RT effectiveness and patient survival across various cancer types. In cervical cancer patients, TT Sims and colleagues demonstrated that gut microbiota diversity serves as an independent indicator of survival for those undergoing chemoradiotherapy (CRT).^183^ Higher microbiome diversity is strongly associated with improved survival outcomes and enhanced immune responses.^183^ Likewise, in patients with NSCLC, changes in gut microbiota composition post‐CRT correlate with progression‐free survival (PFS). Specifically, the presence of *Firmicutes* and higher microbial diversity is linked to longer PFS.^64^ In patients with oesophageal squamous cell carcinoma (ESCC), Sasaki et al.^184^ reported that patients who achieved partial or complete remission from CRT exhibited a significantly higher relative abundance of *Lactobacillaceae*, indicating its viability as a predictive biomarker for CRT efficacy. In CRC patients, elevated levels of *F. nucleatum* and *B. fragilis* are linked to worse survival outcomes.^185^ However, Sánchez‐Alcoholado et al.^76^ suggested that *F. nucleatum* and *B. fragilis* serve as biomarkers for responders to colorectal cancer RCT treatment, whereas *R. gnavus*, *Bifidobacterium bifidum* and *Faecalibacterium prausnitzii* are indicative of non‐responders. A prospective study on microsatellite‐stable colorectal cancer (MSS‐CRC) finds that enrichment of *Lactobacillus* and *Bifidobacterium* predicted better disease control rate and PFS of RT patients treated with fruquintinib and sintilimab.^186^

Specific gut microbiome profiles are strongly linked to the severity of radiation‐induced toxicities and can act as predictive biomarkers for RI. Al‐Qadami et al.^187^ reveal that specific intestinal microbiome profiles are correlated with the intensity of OM in head and neck cancer patients. *Eubacterium* and *Ruminococcus* are linked to more severe OM, while *Faecalibacterium* and *Phascolarctobacterium* are linked to improved treatment results. Furthermore, patients suffering from recurrence exhibit significantly increased relative abundance of *Adlercreutzia* and *Eggerthella lenta*.^187^ These findings highlight potential targets for treating or preventing OM and predicting RT outcomes by modulating the gut flora. In cases of acute radiation‐induced esophagitis (ARIE), Lin et al.^63^ observe that severe ARIE patients have lower alpha diversity and increased levels of *Fusobacterium*. Conversely, mild ARIE patients show enrichment of *Klebsiella*, *Roseburia* and *Veillonella*. In prostate cancer patients, gut microbiota composition predicts acute gastrointestinal toxicity. High‐risk patients exhibit elevated levels of *Bacteroides* and *Sutterella*, whereas low‐risk patients are enriched with *Roseburia* and *Faecalibacterium*.^188^ In cancer patients undergoing pelvic RT, higher abundances of *Bacteroides*, *Ochrobactrum* and *Roseburia* are closely associated with increased gastrointestinal toxicity, while greater abundances of *Clostridia* and *Faecalibacterium* are linked to reduced toxicity.^189^ Similarly, in radiation‐induced liver injury, the abundance of *Romboutsia* correlates with improved survival, suggesting its utility as a predictive biomarker.^56^ Additionally, *Erysipelatoclostridium* and its metabolite ptilosteroid A may serve as biomarkers for predicting RIII.^190^ Emerging research has also highlighted microbial metabolites as potential therapeutic targets. Cyclic‐di‐AMP, a bacterial second messenger, holds promise for predicting and modulating RT efficacy, particularly in HCC patients.^77^

In conclusion, specific microbiome and microbial metabolites characteristics are associated with RT sensitivity or tolerance, supporting the development of microbiome‐based predictive markers. This evidence highlights potential directions for improving therapy and reducing adverse effects in patients undergoing RT.

## 6 CLINICAL APPLICATIONS AND PROSPECTS

In recent years, microbiota‐based therapies such as probiotics, prebiotics, and FMT have emerged as promising strategies to reduce the negative impacts of RT and enhance its effectiveness.^191^ These therapies aim to modulate the gastrointestinal microbiota, acting as a key element in maintaining immune homeostasis, tissue repair, and inflammation regulation, all of which are affected during RT. Among these therapies, FMT plays a particularly crucial role in RT. It helps restore gut microbial balance, reduce RT‐induced toxicity and enhance RT efficacy.^192^ By transferring a healthy microbiota from a donor to a patient, FMT helps restore the microbial diversity that is often disrupted by RT, promoting better immune responses and reducing inflammation. Additionally, FMT has demonstrated effectiveness in preventing and treating RE.^193^ Several case reports have shown that FMT can effectively alleviate clinical symptoms of chronic radiation enteritis (CRE), enhance quality of life, and improve patient prognosis by altering the gut microbiota composition.^194^, ^195^ This shows that FMT not only improves clinical outcomes but also provides significant support for long‐term recovery after RT. Additionally, multiple clinical studies have confirmed that FMT can prevent complications like rectal bleeding, bowel incontinence, diarrhoea, and mucosal injury in CRE patients over time, providing relief from symptoms and improving overall well‐being.^196^

Beyond FMT, probiotics have also shown considerable benefits for RT patients (Table 4). A clinical trial conducted by Linn et al.^197^ showed that RT patients consuming *L. acidophilus* and *Bifidobacterium* experience lower rates of abdominal pain and diarrhoea. This suggests probiotic supplementation as an efficient method to reduce the occurrence and intensity of radiation‐induced diarrhoea in cervical cancer patients.^197^ Another clinical study demonstrates that oral administration of *Bifidobacterium* tablets confers preventive benefits for RT‐induced OM in HSCT.^198^ As demonstrated by a trial conducted by Ahrén et al.,^199^ intake of *L. plantarum* 299 and *L. plantarum* HEAL9 also alleviates gastrointestinal symptoms and reduces radiation toxicity in women receiving pelvic RT for gynaecological cancers. Despite these promising results, clinical research on probiotics remains limited, and many studies examining the benefits of specific probiotic strains in RT patients lack conclusive evidence and robust data. This leaves a notable gap in the field of intestinal flora research, indicating the need for more comprehensive studies and precisely formulated clinical assessments aimed at validating the effectiveness of probiotics and other microbiota‐based therapies in enhancing RT outcomes.

**TABLE 4:** Probiotics and prebiotics in radiotherapy trials.

Study	Disease	Observation endpoint	Patients (*n*)	Intervention	Study status	Results
NCT03870607	Anal cancer	Complete clinical and radiological response after Ch‐RT to ACSCC	75	*Bifidobacterium* and *Lactobacillus*	Recruiting	/
NCT03773003	Cancer	Improvement of fatigue symptoms	150	*Bifidobacterium* spp., *Streptococcus thermophilus* and *Lactobacillus* spp.	Recruiting	/
NCT01839721	Pelvic cancer	The incidence of moderate or severe symptoms of diarrhoea during the period of treatment by RT	246	*Lactobacillus acidophilus LAC‐361* and *Bifidobacterium longum BB‐536*	Completed	Probiotics help reduce diarrhoea rates in patients.
NCT01706393	Gynecologic cancer; rectal cancer	Alterations in gut microbiota in patients with malignancies undergoing pelvic or abdominal RT	26	*Lactobacillus acidophilus, Streptococcus thermophilus, Bifidobacterium lactis, Lactobacillus rhamnosus, Bifidobacterium longum, and Bifidobacterium bifidum*	Unknown	/
NCT03742596	Colorectal cancer	The level of Immunoglobulin (Ig) A at both baseline and end‐line of intervention	40	*Lactobacillus* and *Bifidobacteria*	Withdrawn	/
NCT06390176	Head and neck malignant tumours	The incidence of severe OM (WHO grade ≥3)	132	*Lactobacillus rhamnosus*	Recruiting	/
NCT03112837	Nasopharyngeal cancer	The incidence of radiation therapy oncology group grade 3 mucositis	40	Live combined *Bifidobacterium, Lactobacillus* and *Enterococcus* Capsules	Unknown status	Probiotics lowered OM and protected T cell levels.
NCT01549782	Gynaecologic cancer	Changes in *Lactobacillus* and *Bifidobacterium* populations	40	Mixture of prebiotics: inulin and fructo‐oligosaccharide	Completed	Restore beneficial bacteria

Abbreviations: ACSCC, anal canal squamous cell carcinoma; Ch‐RT, chemoradiotherapy; OM, oral mucositis; RT, radiotherapy.

In addition to these clinically applied therapies, several gut microbiota‐targeted technologies are emerging, offering novel clinical prospects for precision RT and comprehensive cancer treatment. Recent advancements in nanotechnology have opened new avenues for improving RT efficacy and minimizing its side effects. For instance, nanotechnology has advanced cancer treatment by improving RT efficacy and reducing side effects.^200^ Innovations like the bacterial cellulose hydrogel CDDP@SulBC gel enhance gut microbiota balance and radiosensitivity in CRC, demonstrating a promising approach to overcoming RT‐induced gut damage.^201^ Similarly, microcarriers of *Spirulina platensis* combined with Amifostine have been shown to provide comprehensive radioprotection for the small intestine, reducing the toxic effects of RT on intestinal health and improving patient comfort during treatment.^202^

Moreover, studies are exploring the synergistic effects of combining RT with engineered bacteria to enhance immune responses and overall treatment outcomes. A study by Wang et al.^203^, ^204^ shows that combining RT with the injection of antigen‐adsorbed nanoparticle‐coated gene‐deleted *Salmonella* into the tumour can effectively enhance dendritic cell activation and adaptive immune response, significantly improving systemic anti‐tumour effects, and providing a new strategy for in situ cancer vaccination. Additionally, engineered *Salmonella* secreting nattokinase (VNP NKase) enhances the abscopal and immune memory effect of RT, illustrating the potential of clinical implications in combined engineered bacteria therapy with RT to improve cancer patients’ prognosis.^205^

In conclusion, strategies for manipulating the gut microbiota—such as probiotics, prebiotics, postbiotics and FMT—offer effective ways to mitigate RIID and improve cancer prognosis.

## 7 CONCLUSION AND FUTURE DIRECTIONS

The intricate interactions between gut microbiota, their metabolites, and RT influence both therapeutic efficacy and toxicity, underscoring the systemic role of the gut–organ axis across organs like the brain, liver, lungs, skin and the hematopoietic system. Key metabolites, including SCFAs, BAs and tryptophan derivatives, exhibit radioprotective properties and support tissue regeneration, while specific microbial profiles have been linked to RT responses, patient survival and toxicity risks in various cancers.

Despite these advances, significant challenges remain. Clinical evidence for microbiota‐targeted interventions is still limited, constrained by variable patient responses and the lack of standardized protocols. Moreover, the complex interplay between gut microbes and RT‐induced immune and metabolic pathways requires further exploration to bridge laboratory findings with clinical practice.

Future research should focus on identifying microbiota biomarkers, such as *F. nucleatum*, *B. fragilis* and SCFAs, to guide personalized RT strategies. Precision modulation of the intestinal flora through probiotics, prebiotics, dietary adjustments, or FMT could enhance RT outcomes and minimize toxicity. Integrating these approaches with emerging RT technologies like FLASH‐RT may further protect healthy tissues and improve tumour radiosensitivity. Investigating the radioprotective roles of microbial metabolites could also unlock new therapeutic approaches.

Standardized methodologies and large‐scale clinical trials involving diverse populations are essential to validate these interventions. Simultaneously, deeper exploration of the gut–organ axis, particularly its role in shielding distant organs from RT‐induced damage, is critical for addressing conditions like radiation‐induced brain injury, liver toxicity, and pulmonary fibrosis. Incorporating artificial intelligence and multi‐omics technologies, such as metagenomics and metabolomics, can deepen our understanding of microbiota–RT interactions and help identify novel biomarkers and therapeutic targets. Long‐term monitoring of microbiota changes before, during, and after RT can provide insights into its enduring impact on gut health and systemic recovery, guiding post‐treatment strategies.

In summary, targeting gut microbiota and their metabolites represents a transformative approach to optimizing RT. By integrating microbiota research with clinical applications, we can usher in an era of personalized cancer treatment that maximizes therapeutic benefits while minimizing adverse effects. Continued multidisciplinary collaboration will be key to unlocking the full potential of this promising field.

## AUTHOR CONTRIBUTIONS

**Shuling Ma**: Writing—original draft; visualization. **Xinpei Li**: Writing—review & editing. **Shijie Shang**: Writing—review & editing. **Zijun Zhai**: Writing—review & editing. **Meng Wu**: Supervision. **Qian Song**: Writing—review & editing; supervision; conceptualization. **Dawei Chen**: Writing—review & editing; supervision; project administration.

## CONFLICT OF INTEREST STATEMENT

The authors declare no conflict of interest.

## FUNDING STATEMENT

This work was funded by the National Natural Science Foundation of China (82172676, 82373217, and 82403455), the Natural Science Foundation of Shandong (ZR2024QH527), the Distinguished Young Scholars of Shandong Provincial Science Fund (ZR2024JQ032), Shandong Province International Science and Technology Cooperation Project (2025KJHZ012), the China Postdoctoral Science Foundation (2024M761869 and 2025T180617).

## ETHICS APPROVAL STATEMENT

Not applicable.

## PERMISSION TO REPRODUCE MATERIAL FROM OTHER SOURCES

Not applicable.

## Data Availability

Data sharing is not applicable to this article as no new data were created or analyzed in this study.
